# Supplementary material for: Kynurenines and aerobic exercise capacity in chronic kidney disease: A cross-sectional and longitudinal study
Source: PLoS One. 2025 Jan 15;20(1):e0317201. doi: 10.1371/journal.pone.0317201 (PMC11734918; doi:10.1371/journal.pone.0317201)
Supplement: S1 Table — (DOCX) [file pone.0317201.s001.docx]

**S1 Table.** **Association between aerobic exercise capacity and kynurenines, TRP and GFR in CKD 2–5 at baseline.**

| **Dependent variable** Exercise capacity | **Independent Variable** | **Beta** | **Stand. Beta** | **p-value** | **n** | **R^2^** |
| --- | --- | --- | --- | --- | --- | --- |
|  | Age  Female sex  Height^2^ | –1.52  –55  44 |  | < 0.001  < 0.001  0.09 | 98 | 0.458 |
| Model 1  Exercise capacity | KYN | –1.80 | –0.05 | 0.5 | 97 | 0.457 |
| Model 2  Exercise capacity | KYNA | –0.042 | –0.14 | 0.02 | 96 | 0.477 |
| Model 3  Exercise capacity | TRP | 0.45 | 0.14 | 0.07 | 92 | 0.475 |
| Model 4  Exercise capacity | KYN/TRP | –131 | –0.17 | 0.01 | 92 | 0.473 |
| Model 5  Exercise capacity | KYNA/KYN | –0.27 | –0.14 | 0.02 | 96 | 0.478 |
| Model 6  Exercise capacity | GFR | 0.73 | 0.26 | < 0.001 | 98 | 0.528 |

n = number of subjects. Assessed by GLM (generalised linear model. Age, sex and height included in all models. Beta and p-values for age, sex and height changes in the different models. R^2^ was calculated in a linear regression model, including age, sex and height^2^ KYN = kynurenine, KYNA = kynurenic acid, TRP = tryptophan, GFR = glomerular filtration rate, Beta = unstandardised beta coefficient; Stand. Beta = standardised beta coefficient.
