# Supplementary material for: Kynurenines and aerobic exercise capacity in chronic kidney disease: A cross-sectional and longitudinal study
Source: PLoS One. 2025 Jan 15;20(1):e0317201. doi: 10.1371/journal.pone.0317201 (PMC11734918; doi:10.1371/journal.pone.0317201)
Supplement: S2 Table — (DOCX) [file pone.0317201.s002.docx]

**S2 Table. Predictors of the change in aerobic exercise capacity from baseline to 5-year follow-up in CKD 2–3.**

|  | **Variable** | **Beta** | **Stand. beta** | **p-value** | **n** |
| --- | --- | --- | --- | --- | --- |
|  | Delta KYN | –3.8 | –0.30 | 0.003 | 44 |
|  | Delta KYNA | –0.1 | –0.20 | 0.06 | 41 |
|  | Delta TRP | 0.085 | 0.09 | 0.6 | 42 |
|  | Delta KYN/TRP | –246 | –0.54 | < 0.001 | 42 |
|  | Delta KYNA/KYN | 0.05 | 0.02 | 0.9 | 41 |
|  | Delta GFR | 0.45 | 0.34 | 0.005 | 42 |
|  |  | | | | |
| Model 1 | Delta GFR | 0.33 | 0.25 | 0.03 | 41 |
|  | Delta KYN | –2.94 | –0.23 | 0.05 |  |
| Model 2 | Delta GFR | 0.16 | 0.12 | 0.3 | 38 |
|  | Delta KYNA | –0.08 | –0.15 | 0.3 |  |
| Model 3 | Delta GFR | 0.23 | 0.18 | 0.2 | 39 |
|  | Delta KYN/TRP | –197 | –0.43 | 0.009 |  |

n = number of subjects. Delta value = 5-year follow-up – baseline. Assessed by GLM (generalised linear model). KYN = kynurenine, KYNA = kynurenic acid, TRP = tryptophan. Beta = unstandardised beta coefficient; Stand. beta = standardised beta coefficient.
