# Supplementary material for: Kynurenines and aerobic exercise capacity in chronic kidney disease: A cross-sectional and longitudinal study
Source: PLoS One. 2025 Jan 15;20(1):e0317201. doi: 10.1371/journal.pone.0317201 (PMC11734918; doi:10.1371/journal.pone.0317201)
Supplement: S1 File — (PDF) [file pone.0317201.s003.pdf]

Oneway

Notes

Output Created		23-FEB-2024 11:26:16
Comments		
Input	Data	/Users/helena.wallin/Library/CloudStorage/OneDrive-KarolinskaInstitutet/Copied_from_Box/Helenas arbetsdokument/Aerobic capacity in CKD/Progress Ex/Databaser/spss/Kyn 2021/Progress5yearNov 2021WITHoutliers.sav
	Active Dataset	DataSet1
	Filter	<none>
	Weight	<none>
	Split File	<none>
	N of Rows in Working Data File	179
Missing Value Handling	Definition of Missing	User-defined missing values are treated as missing.
	Cases Used	Statistics for each analysis are based on cases with no missing data for any variable in the analysis.
Syntax		ONEWAY kyn0 kyna0 trp0 nykvot0 KynaKyn0 BY Grupp<br>/ES=OVERALL<br>/STATISTICS DESCRIPTIVES HOMOGENEITY WELCH<br>/MISSING ANALYSIS<br>/CRITERIA=CILEVEL (0.95)<br>/POSTHOC=T3 ALPHA (0.05).
Resources	Processor Time	00:00:00,05
	Elapsed Time	00:00:00,00

Descriptives

		N	Mean	Std. Deviation	Std. Error	95% Confidence ...
						Lower Bound
kyn0	1	48	6.0891	1.77999	.25692	5.5722
	2	53	3.7509	1.12157	.15406	3.4418
	3	53	2.7073	.90634	.12450	2.4575
	Total	154	4.1205	1.90653	.15363	3.8170
kyna0	1	48	375.8061	230.93881	33.33315	308.7485
	2	53	95.0603	71.91254	9.87795	75.2387
	3	53	58.9034	19.36137	2.65949	53.5667
	Total	154	170.1219	194.36396	15.66229	139.1796
trp0	1	48	52.9865	56.35044	8.13349	36.6240
	2	53	59.0749	35.54707	4.88277	49.2769
	3	53	66.0693	25.68076	3.52752	58.9908
	Total	154	59.5844	40.71065	3.28056	53.1033
nykvot0	1	48	.165572	.0804426	.0116109	.142214
	2	53	.073250	.0339805	.0046676	.063884
	3	53	.043765	.0146693	.0020150	.039721
	Total	154	.091878	.0712691	.0057430	.080532
KynaKyn0	1	48	63.8928	38.45321	5.55024	52.7271
	2	53	24.9754	11.32780	1.55599	21.8531
	3	53	22.3109	5.71839	.78548	20.7348
	Total	154	36.1885	29.32595	2.36315	31.5199

Descriptives

		95% Confidence Interval for ...		
		Upper Bound	Minimum	Maximum
kyn0	1	6.6059	1.90	10.28
	2	4.0601	1.98	7.28
	3	2.9571	1.49	7.34
	Total	4.4241	1.49	10.28
kyna0	1	442.8637	19.82	1052.74
	2	114.8818	41.87	568.72
	3	64.2400	24.05	110.93
	Total	201.0641	19.82	1052.74
trp0	1	69.3489	14.38	301.71
	2	68.8729	24.98	281.53
	3	73.1478	30.84	206.51
	Total	66.0654	14.38	301.71
nykvot0	1	.188930	.0160	.3641
	2	.082617	.0147	.2405
	3	.047808	.0189	.0915
	Total	.103224	.0147	.3641
KynaKyn0	1	75.0584	2.47	180.96
	2	28.0978	8.77	94.32
	3	23.8871	9.93	36.91
	Total	40.8571	2.47	180.96

Tests of Homogeneity of Variances

		Levene Statistic	df1	df2	Sig.
kyn0	Based on Mean	13.307	2	151	<.001
	Based on Median	12.716	2	151	<.001
	Based on Median and with adjusted df	12.716	2	138.409	<.001
	Based on trimmed mean	13.567	2	151	<.001
kyna0	Based on Mean	52.235	2	151	<.001
	Based on Median	43.254	2	151	<.001
	Based on Median and with adjusted df	43.254	2	66.163	<.001
	Based on trimmed mean	49.028	2	151	<.001
trp0	Based on Mean	2.294	2	151	.104
	Based on Median	.749	2	151	.475
	Based on Median and with adjusted df	.749	2	97.447	.476
	Based on trimmed mean	1.005	2	151	.368
nykvot0	Based on Mean	32.960	2	151	<.001
	Based on Median	30.756	2	151	<.001
	Based on Median and with adjusted df	30.756	2	73.243	<.001
	Based on trimmed mean	32.557	2	151	<.001
KynaKyn0	Based on Mean	40.137	2	151	<.001
	Based on Median	32.634	2	151	<.001
	Based on Median and with adjusted df	32.634	2	62.202	<.001
	Based on trimmed mean	36.170	2	151	<.001

ANOVA

		Sum of Squares	df	Mean Square	F	Sig.
kyn0	Between Groups	299.094	2	149.547	87.852	<.001
	Within Groups	257.040	151	1.702		
	Total	556.135	153			
kyna0	Between Groups	2984889.433	2	1492444.716	80.628	<.001
	Within Groups	2795044.891	151	18510.231		
	Total	5779934.324	153			
trp0	Between Groups	4332.166	2	2166.083	1.312	.272
	Within Groups	249243.477	151	1650.619		
	Total	253575.643	153			
nykvot0	Between Groups	.402	2	.201	80.808	<.001
	Within Groups	.375	151	.002		
	Total	.777	153			
KynaKyn0	Between Groups	53712.210	2	26856.105	52.078	<.001
	Within Groups	77869.529	151	515.692		
	Total	131581.739	153			

ANOVA Effect Sizes[a,b]

		Point Estimate	95% Confidence Interval Lower	95% Confidence Interval Upper
kyn0	Eta-squared	.538	.428	.615
	Epsilon-squared	.532	.420	.610
	Omega-squared Fixed-effect	.530	.419	.608
	Omega-squared Random-effect	.361	.265	.437
kyna0	Eta-squared	.516	.404	.596
	Epsilon-squared	.510	.396	.591
	Omega-squared Fixed-effect	.508	.394	.589
	Omega-squared Random-effect	.341	.246	.418
trp0	Eta-squared	.017	.000	.069
	Epsilon-squared	.004	-.013	.056
	Omega-squared Fixed-effect	.004	-.013	.056
	Omega-squared Random-effect	.002	-.007	.029
nykvot0	Eta-squared	.517	.404	.597
	Epsilon-squared	.511	.397	.591
	Omega-squared Fixed-effect	.509	.395	.590
	Omega-squared Random-effect	.341	.246	.418
KynaKyn0	Eta-squared	.408	.286	.501
	Epsilon-squared	.400	.277	.494
	Omega-squared Fixed-effect	.399	.276	.492
	Omega-squared Random-effect	.249	.160	.326

a. Eta-squared and Epsilon-squared are estimated based on the fixed-effect model.

b. Negative but less biased estimates are retained, not rounded to zero.

Robust Tests of Equality of Means

		Statistic[a]	df1	df2	Sig.
kyn0	Welch	71.848	2	92.612	<.001
kyna0	Welch	50.009	2	70.431	<.001
trp0	Welch	1.431	2	90.543	.244
nykvot0	Welch	65.910	2	76.993	<.001
KynaKyn0	Welch	27.842	2	78.853	<.001

a. Asymptotically F distributed.

Post Hoc Tests

Multiple Comparisons

Dunnett T3

Dependent Variable	(I) Grupp	(J) Grupp	Mean Difference (I-J)	Std. Error	Sig.	95% ... Lower Bound
kyn0	1	2	2.33812*	.29957	<.001	1.6077
		3	3.38174*	.28549	.000	2.6836
	2	1	-2.33812*	.29957	<.001	-3.0686
		3	1.04361*	.19807	<.001	.5629
	3	1	-3.38174*	.28549	.000	-4.0799
		2	-1.04361*	.19807	<.001	-1.5244
kyna0	1	2	280.74582*	34.76597	<.001	195.2419
		3	316.90274*	33.43907	<.001	234.2673
	2	1	-280.74582*	34.76597	<.001	-366.2497
		3	36.15692*	10.22970	.002	11.0516
	3	1	-316.90274*	33.43907	<.001	-399.5382
		2	-36.15692*	10.22970	.002	-61.2622
trp0	1	2	-6.08841	9.48657	.890	-29.2190
		3	-13.08280	8.86550	.372	-34.7954
	2	1	6.08841	9.48657	.890	-17.0422
		3	-6.99439	6.02369	.573	-21.6268
	3	1	13.08280	8.86550	.372	-8.6298
		2	6.99439	6.02369	.573	-7.6381
nykvot0	1	2	.0923217*	.0125140	<.001	.061644
		3	.1218076*	.0117844	<.001	.092731
	2	1	-.0923217*	.0125140	<.001	-.122999
		3	.0294859*	.0050839	<.001	.017064
	3	1	-.1218076*	.0117844	<.001	-.150884
		2	-.0294859*	.0050839	<.001	-.041908
KynaKyn0	1	2	38.91733*	5.76423	<.001	24.7340
		3	41.58183*	5.60555	<.001	27.7419
	2	1	-38.91733*	5.76423	<.001	-53.1006
		3	2.66450	1.74301	.340	-1.5865
	3	1	-41.58183*	5.60555	<.001	-55.4218
		2	-2.66450	1.74301	.340	-6.9155

Multiple Comparisons

Dunnett T3

Dependent Variable	(I) Grupp	(J) Grupp	95% ... Upper Bound
kyn0	1	2	3.0686
		3	4.0799
	2	1	-1.6077
		3	1.5244
	3	1	-2.6836
		2	-.5629
kyna0	1	2	366.2497
		3	399.5382
	2	1	-195.2419
		3	61.2622
	3	1	-234.2673
		2	-11.0516
trp0	1	2	17.0422
		3	8.6298
	2	1	29.2190
		3	7.6381
	3	1	34.7954
		2	21.6268
nykvot0	1	2	.122999
		3	.150884
	2	1	-.061644
		3	.041908
	3	1	-.092731
		2	-.017064
KynaKyn0	1	2	53.1006
		3	55.4218
	2	1	-24.7340
		3	6.9155
	3	1	-27.7419
		2	1.5865

*. The mean difference is significant at the 0.05 level.

Oneway

Notes

Output Created		23-FEB-2024 16:12:35
Comments		
Input	Data	/Users/helena.wallin/Library/CloudStorage/OneDrive-KarolinskaInstitutet/Copied_from_Box/Helenas arbetsdokument/Aerobic capacity in CKD/Progress Ex/Databaser/spss/Kyn 2021/Progress5yearNov 2021WITHoutliers.sav
	Active Dataset	DataSet1
	Filter	<none>
	Weight	<none>
	Split File	<none>
	N of Rows in Working Data File	179
Missing Value Handling	Definition of Missing	User-defined missing values are treated as missing.
	Cases Used	Statistics for each analysis are based on cases with no missing data for any variable in the analysis.
Syntax		ONEWAY MaxWBL BY Grupp<br>/ES=OVERALL<br>/STATISTICS DESCRIPTIVES<br>/MISSING ANALYSIS<br>/CRITERIA=CILEVEL(0.95)<br>/POSTHOC=TUKEY ALPHA(0.05).
Resources	Processor Time	00:00:00,02
	Elapsed Time	00:00:00,00

Descriptives

MaxWBL

	N	Mean	Std. Deviation	Std. Error	95% Confidence Interval for Mean	
					Lower Bound	Upper Bound
1	47	155.64	57.451	8.380	138.77	172.51
2	52	193.08	62.694	8.694	175.62	210.53
3	54	237.50	59.801	8.138	221.18	253.82
Total	153	197.25	68.422	5.532	186.33	208.18

Descriptives

MaxWBL

	Minimum	Maximum
1	60	280
2	80	340
3	140	410
Total	60	410

ANOVA

MaxWBL

	Sum of Squares	df	Mean Square	F	Sig.
Between Groups	169771.015	2	84885.508	23.500	<.001
Within Groups	541826.043	150	3612.174		
Total	711597.059	152			

ANOVA Effect Sizes[a]

		Point Estimate	95% Confidence Interval	
			Lower	Upper
MaxWBL	Eta-squared	.239	.124	.341
	Epsilon-squared	.228	.112	.332
	Omega-squared Fixed-effect	.227	.111	.330
	Omega-squared Random-effect	.128	.059	.198

a. Eta-squared and Epsilon-squared are estimated based on the fixed-effect model.

Post Hoc Tests

Multiple Comparisons

Dependent Variable: MaxWBL

Tukey HSD

(I) Grupp	(J) Grupp	Mean Difference (I-J)	Std. Error	Sig.	95% Confidence Interval	
					Lower Bound	Upper Bound
1	2	-37.439*	12.096	.007	-66.07	-8.80
	3	-81.862*	11.989	<.001	-110.24	-53.48
2	1	37.439*	12.096	.007	8.80	66.07
	3	-44.423*	11.677	<.001	-72.07	-16.78
3	1	81.862*	11.989	<.001	53.48	110.24
	2	44.423*	11.677	<.001	16.78	72.07

*. The mean difference is significant at the 0.05 level.

Homogeneous Subsets

MaxWBL

Tukey HSD[a,b]

Grupp	N	Subset for alpha = 0.05		
		1	2	3
1	47	155.64		
2	52		193.08	
3	54			237.50
Sig.		1.000	1.000	1.000

Means for groups in homogeneous subsets are displayed.

a. Uses Harmonic Mean Sample Size = 50,825.

b. The group sizes are unequal. The harmonic mean of the group sizes is used. Type I error levels are not guaranteed.

T-Test

Notes

Output Created		23-FEB-2024 16:16:24
Comments		
Input	Data	/Users/helena.wallin/Library/CloudStorage/OneDrive-KarolinskaInstitutet/Copied_from_Box/Helenas arbetsdokument/Aerobic capacity in CKD/Progress Ex/Databaser/spss/Kyn 2021/Progress5yearNov2021WITHoutliers.sav
	Active Dataset	DataSet1
	Filter	Grupp = 2 (FILTER)
	Weight	<none>
	Split File	<none>
	N of Rows in Working Data File	54
Missing Value Handling	Definition of Missing	User defined missing values are treated as missing.
	Cases Used	Statistics for each analysis are based on the cases with no missing or out-of-range data for any variable in the analysis.

Notes

Syntax		T-TEST PAIRS=kyn0 trp0 nykvot0 KynaKyn0 CKDepi0 HbBL MaxWBL kyna0 WITH kyn5 trp5 nykvot5 KynaKyn5 CKDepi5 HB5 MaxW5 kyna5 (PAIRED) /ES DISPLAY(TRUE) STANDARDIZER(SD) /CRITERIA=CI(.9500) /MISSING=ANALYSIS.
Resources	Processor Time	00:00:00,03
	Elapsed Time	00:00:00,00

Paired Samples Statistics

		Mean	N	Std. Deviation	Std. Error Mean
Pair 1	kyn0	3.7121	47	1.15816	.16894
	kyn5	3.9920	47	1.27440	.18589
Pair 2	trp0	61.1195	47	37.12922	5.41585
	trp5	63.5307	47	13.99770	2.04177
Pair 3	nykvot0	.070054	47	.0338870	.0049429
	nykvot5	.065751	47	.0268331	.0039140
Pair 4	KynaKyn0	25.5425	47	11.77237	1.71718
	KynaKyn5	28.8594	47	15.60685	2.27649
Pair 5	CKDepi0	58.48	48	13.418	1.937
	CKDepi5	49.56	48	16.823	2.428
Pair 6	HbBL	135.34	50	13.940	1.971
	HB5	138.68	50	12.961	1.833
Pair 7	MaxWBL	198.89	45	60.301	8.989
	MaxW5	193.18	45	61.113	9.110
Pair 8	kyna0	96.5262	47	75.60865	11.02866
	kyna5	113.6913	47	77.02882	11.23581

Paired Samples Correlations

		N	Correlation	Significance	
				One-Sided p	Two-Sided p
Pair 1	kyn0 & kyn5	47	.175	.119	.239
Pair 2	trp0 & trp5	47	-.019	.449	.897
Pair 3	nykvot0 & nykvot5	47	-.101	.250	.500
Pair 4	KynaKyn0 & KynaKyn5	47	.143	.168	.336
Pair 5	CKDepi0 & CKDepi5	48	.558	<.001	<.001
Pair 6	HbBL & HB5	50	.647	<.001	<.001
Pair 7	MaxWBL & MaxW5	45	.946	<.001	<.001
Pair 8	kyna0 & kyna5	47	.037	.402	.803

Paired Samples Test

		Paired Differences			
					95% Confidence ...
		Mean	Std. Deviation	Std. Error Mean	Lower
Pair 1	kyn0 - kyn5	-.27994	1.56467	.22823	-.73934
Pair 2	trp0 - trp5	-2.41122	39.93298	5.82482	-14.13598
Pair 3	nykvot0 - nykvot5	.0043032	.0452982	.0066074	-.0089969
Pair 4	KynaKyn0 - KynaKyn5	-3.31689	18.15067	2.64755	-8.64613
Pair 5	CKDepi0 - CKDepi5	8.917	14.534	2.098	4.697
Pair 6	HbBL - HB5	-3.340	11.338	1.603	-6.562
Pair 7	MaxWBL - MaxW5	5.711	19.981	2.979	-.292
Pair 8	kyna0 - kyna5	-17.16508	105.90286	15.44752	-48.25932

Paired Samples Test

		Paired ...			Significance
		95% Confidence Interval of the...			
		Upper	t	df	One-Sided p
Pair 1	kyn0 - kyn5	.17946	-1.227	46	.113
Pair 2	trp0 - trp5	9.31354	-.414	46	.340
Pair 3	nykvot0 - nykvot5	.0176032	.651	46	.259
Pair 4	KynaKyn0 - KynaKyn5	2.01235	-1.253	46	.108
Pair 5	CKDepi0 - CKDepi5	13.137	4.251	47	<.001
Pair 6	HbBL - HB5	-.118	-2.083	49	.021
Pair 7	MaxWBL - MaxW5	11.714	1.917	44	.031
Pair 8	kyna0 - kyna5	13.92917	-1.111	46	.136

Paired Samples Test

		Significance
		Two-Sided p
Pair 1	kyn0 - kyn5	.226
Pair 2	trp0 - trp5	.681
Pair 3	nykvot0 - nykvot5	.518
Pair 4	KynaKyn0 - KynaKyn5	.217
Pair 5	CKDepi0 - CKDepi5	<.001
Pair 6	HbBL - HB5	.042
Pair 7	MaxWBL - MaxW5	.062
Pair 8	kyna0 - kyna5	.272

Paired Samples Effect Sizes

			Standardizer[a]	Point Estimate	95% ... Lower
Pair 1	kyn0 - kyn5	Cohen's d	1.56467	-.179	-.466
		Hedges' correction	1.59077	-.176	-.459
Pair 2	trp0 - trp5	Cohen's d	39.93298	-.060	-.346
		Hedges' correction	40.59915	-.059	-.341
Pair 3	nykvot0 - nykvot5	Cohen's d	.0452982	.095	-.192
		Hedges' correction	.0460539	.093	-.189
Pair 4	KynaKyn0 - KynaKyn5	Cohen's d	18.15067	-.183	-.470
		Hedges' correction	18.45346	-.180	-.462
Pair 5	CKDepi0 - CKDepi5	Cohen's d	14.534	.614	.302
		Hedges' correction	14.771	.604	.297
Pair 6	HbBL - HB5	Cohen's d	11.338	-.295	-.576
		Hedges' correction	11.516	-.290	-.567
Pair 7	MaxWBL - MaxW5	Cohen's d	19.981	.286	-.014
		Hedges' correction	20.330	.281	-.014
Pair 8	kyna0 - kyna5	Cohen's d	105.90286	-.162	-.449
		Hedges' correction	107.66955	-.159	-.442

Paired Samples Effect Sizes

			95% ... Upper
Pair 1	kyn0 - kyn5	Cohen's d	.110
		Hedges' correction	.108
Pair 2	trp0 - trp5	Cohen's d	.226
		Hedges' correction	.222
Pair 3	nykvot0 - nykvot5	Cohen's d	.381
		Hedges' correction	.375
Pair 4	KynaKyn0 - KynaKyn5	Cohen's d	.107
		Hedges' correction	.105
Pair 5	CKDepi0 - CKDepi5	Cohen's d	.920
		Hedges' correction	.905
Pair 6	HbBL - HB5	Cohen's d	-.010
		Hedges' correction	-.010
Pair 7	MaxWBL - MaxW5	Cohen's d	.582
		Hedges' correction	.572
Pair 8	kyna0 - kyna5	Cohen's d	.127
		Hedges' correction	.124

a. The denominator used in estimating the effect sizes.
Cohen's d uses the sample standard deviation of the mean difference.
Hedges' correction uses the sample standard deviation of the mean difference, plus a correction factor.

```
USE ALL.
COMPUTE filter_$=(Grupp = 2).
VARIABLE LABELS filter_$ 'Grupp = 2 (FILTER)'.
VALUE LABELS filter_$ 0 'Not Selected' 1 'Selected'.
```

```
FORMATS filter_$ (f1.0).
FILTER BY filter_$.
EXECUTE.
T-TEST PAIRS=kyn0 kyna0 trp0 nykvot0 KynaKyn0 WITH kyn5 kyna5 trp5 nykvot5 KynaKyn5
(PAIRED)
/ES DISPLAY(TRUE) STANDARDIZER(SD)
/CRITERIA=CI(.9500)
/MISSING=ANALYSIS.


USE ALL.
COMPUTE filter_$=(Grupp = 3).
VARIABLE LABELS filter_$ 'Grupp = 3 (FILTER)'.
VALUE LABELS filter_$ 0 'Not Selected' 1 'Selected'.
FORMATS filter_$ (f1.0).
FILTER BY filter_$.
EXECUTE.
T-TEST PAIRS=kyn0 trp0 nykvot0 KynaKyn0 CKDepi0 HbBL MaxWBL kyna0 WITH kyn5 trp5
nykvot5 KynaKyn5
CKDepi5 HB5 MaxW5 kyna5 (PAIRED)
/ES DISPLAY(TRUE) STANDARDIZER(SD)
/CRITERIA=CI(.9500)
/MISSING=ANALYSIS.
```

T-Test

Notes

Output Created		23-FEB-2024 19:46:53
Comments		
Input	Data	/Users/helena.wallin/Library/CloudStorage/OneDrive-KarolinskaInstitutet/Copied_from_Box/Helenas arbetsdokument/Aerobic capacity in CKD/Progress Ex/Databaser/spss/Kyn 2021/Progress5yearNov 2021WITHoutliers.sav
	Active Dataset	DataSet1
	Filter	Grupp = 3 (FILTER)
	Weight	<none>
	Split File	<none>
	N of Rows in Working Data File	54
Missing Value Handling	Definition of Missing	User defined missing values are treated as missing.
	Cases Used	Statistics for each analysis are based on the cases with no missing or out-of-range data for any variable in the analysis.

Notes

Syntax		T-TEST PAIRS=kyn0 trp0 nykvot0 KynaKyn0 CKDepi0 HbBL MaxWBL kyna0 WITH kyn5 trp5 nykvot5 KynaKyn5 CKDepi5 HB5 MaxW5 kyna5 (PAIRED) /ES DISPLAY(TRUE) STANDARDIZER(SD) /CRITERIA=CI(.9500) /MISSING=ANALYSIS.
Resources	Processor Time	00:00:00,04
	Elapsed Time	00:00:00,00

Paired Samples Statistics

		Mean	N	Std. Deviation	Std. Error Mean
Pair 1	kyn0	2.7868	41	.99355	.15517
	kyn5	2.6351	41	.51714	.08076
Pair 2	trp0	67.4872	41	27.83662	4.34735
	trp5	67.8739	41	11.48064	1.79297
Pair 3	nykvot0	.044241	41	.0153321	.0023945
	nykvot5	.039397	41	.0078785	.0012304
Pair 4	KynaKyn0	22.1140	41	5.84032	.91210
	KynaKyn5	22.4017	41	5.27138	.82325
Pair 5	CKDepi0	94.19	43	11.931	1.819
	CKDepi5	90.67	43	11.224	1.712
Pair 6	HbBL	142.35	40	11.235	1.776
	HB5	147.48	40	10.751	1.700
Pair 7	MaxWBL	245.50	40	63.162	9.987
	MaxW5	240.93	40	69.711	11.022
Pair 8	kyna0	59.5521	41	19.16088	2.99243
	kyna5	58.5905	41	16.08622	2.51225

Paired Samples Correlations

		N	Correlation	Significance	
				One-Sided p	Two-Sided p
Pair 1	kyn0 & kyn5	41	.193	.113	.226
Pair 2	trp0 & trp5	41	.131	.206	.413
Pair 3	nykvot0 & nykvot5	41	.536	<.001	<.001
Pair 4	KynaKyn0 & KynaKyn5	41	.328	.018	.037
Pair 5	CKDepi0 & CKDepi5	43	.777	<.001	<.001
Pair 6	HbBL & HB5	40	.618	<.001	<.001
Pair 7	MaxWBL & MaxW5	40	.912	<.001	<.001
Pair 8	kyna0 & kyna5	41	.534	<.001	<.001

Paired Samples Test

		Paired Differences			
					95% Confidence ...
		Mean	Std. Deviation	Std. Error Mean	Lower
Pair 1	kyn0 - kyn5	.15168	1.02763	.16049	-.17268
Pair 2	trp0 - trp5	-.38675	28.68261	4.47947	-9.44009
Pair 3	nykvot0 - nykvot5	.0048432	.0129523	.0020228	.0007550
Pair 4	KynaKyn0 - KynaKyn5	-.28774	6.45932	1.00878	-2.32655
Pair 5	CKDepi0 - CKDepi5	3.512	7.759	1.183	1.124
Pair 6	HbBL - HB5	-5.125	9.616	1.520	-8.200
Pair 7	MaxWBL - MaxW5	4.575	28.584	4.519	-4.566
Pair 8	kyna0 - kyna5	.96164	17.23277	2.69131	-4.47769

Paired Samples Test

		Paired ...			Significance
		95% Confidence Interval of the...			
		Upper	t	df	One-Sided p
Pair 1	kyn0 - kyn5	.47604	.945	40	.175
Pair 2	trp0 - trp5	8.66660	-.086	40	.466
Pair 3	nykvot0 - nykvot5	.0089315	2.394	40	.011
Pair 4	KynaKyn0 - KynaKyn5	1.75107	-.285	40	.388
Pair 5	CKDepi0 - CKDepi5	5.900	2.968	42	.002
Pair 6	HbBL - HB5	-2.050	-3.371	39	<.001
Pair 7	MaxWBL - MaxW5	13.716	1.012	39	.159
Pair 8	kyna0 - kyna5	6.40097	.357	40	.361

Paired Samples Test

		Significance
		Two-Sided p
Pair 1	kyn0 - kyn5	.350
Pair 2	trp0 - trp5	.932
Pair 3	nykvot0 - nykvot5	.021
Pair 4	KynaKyn0 - KynaKyn5	.777
Pair 5	CKDepi0 - CKDepi5	.005
Pair 6	HbBL - HB5	.002
Pair 7	MaxWBL - MaxW5	.318
Pair 8	kyna0 - kyna5	.723

Paired Samples Effect Sizes

			Standardizer[a]	Point Estimate	95% … Lower
Pair 1	kyn0 - kyn5	Cohen's d	1.02763	.148	-.161
		Hedges' correction	1.04741	.145	-.158
Pair 2	trp0 - trp5	Cohen's d	28.68261	-.013	-.320
		Hedges' correction	29.23479	-.013	-.313
Pair 3	nykvot0 - nykvot5	Cohen's d	.0129523	.374	.055
		Hedges' correction	.0132017	.367	.054
Pair 4	KynaKyn0 - KynaKyn5	Cohen's d	6.45932	-.045	-.351
		Hedges' correction	6.58367	-.044	-.344
Pair 5	CKDepi0 - CKDepi5	Cohen's d	7.759	.453	.136
		Hedges' correction	7.901	.444	.134
Pair 6	HbBL - HB5	Cohen's d	9.616	-.533	-.862
		Hedges' correction	9.806	-.523	-.845
Pair 7	MaxWBL - MaxW5	Cohen's d	28.584	.160	-.153
		Hedges' correction	29.148	.157	-.150
Pair 8	kyna0 - kyna5	Cohen's d	17.23277	.056	-.251
		Hedges' correction	17.56452	.055	-.246

Paired Samples Effect Sizes

			95% … Upper
Pair 1	kyn0 - kyn5	Cohen's d	.454
		Hedges' correction	.446
Pair 2	trp0 - trp5	Cohen's d	.293
		Hedges' correction	.287
Pair 3	nykvot0 - nykvot5	Cohen's d	.689
		Hedges' correction	.676
Pair 4	KynaKyn0 - KynaKyn5	Cohen's d	.262
		Hedges' correction	.257
Pair 5	CKDepi0 - CKDepi5	Cohen's d	.764
		Hedges' correction	.750
Pair 6	HbBL - HB5	Cohen's d	-.198
		Hedges' correction	-.195
Pair 7	MaxWBL - MaxW5	Cohen's d	.471
		Hedges' correction	.462
Pair 8	kyna0 - kyna5	Cohen's d	.362
		Hedges' correction	.355

a. The denominator used in estimating the effect sizes.
Cohen's d uses the sample standard deviation of the mean difference.
Hedges' correction uses the sample standard deviation of the mean difference, plus a correction factor.
