# Supplementary material for: Kynurenines and aerobic exercise capacity in chronic kidney disease: A cross-sectional and longitudinal study
Source: PLoS One. 2025 Jan 15;20(1):e0317201. doi: 10.1371/journal.pone.0317201 (PMC11734918; doi:10.1371/journal.pone.0317201)
Supplement: Attachment — Submitted filename: PONE-D-24-08103 Cr.pdf [file pone.0317201.s005.pdf]

PONE-D-24-08103

Kynurenines and aerobic exercise capacity in chronic kidney disease: a cross-sectional and longitudinal study

This is an interesting cross-sectional and longitudinal study, properly conducted, that analyzes the association between aerobic exercise capacity and kynurenines in non-dialysis patients with chronic kidney disease, aimed to show evidence that aerobic exercise capacity would be negatively associated with plasma levels of kynurenine, kynurenine/amino acid tryptophan and kynurenic acid in chronic kidney disease.

However, the relevance of these results in the clinic in patients is still underway. Furthermore, it has not been taken in account some other different or additional comorbidities to chronic kidney disease.

Nevertheless, this study may establish an interesting basis or support to future analysis in this sense, including studies on mitochondrial dysfunction, as suggested by the authors themselves, and/or potential molecular mechanisms involved in these processes.
