# Supplementary material for: Kynurenines and aerobic exercise capacity in chronic kidney disease: A cross-sectional and longitudinal study
Source: PLoS One. 2025 Jan 15;20(1):e0317201. doi: 10.1371/journal.pone.0317201 (PMC11734918; doi:10.1371/journal.pone.0317201)
Supplement: Attachment — Submitted filename: Response to reviewers 241220.docx [file pone.0317201.s007.docx]

**Authors response to reviewers and editor:**

Thank you for the feedback. We have provided our response to reviewer 4 below under the reviewers’ comments.

Reviewer's Responses to Questions

**Comments to the Author**

1. If the authors have adequately addressed your comments raised in a previous round of review and you feel that this manuscript is now acceptable for publication, you may indicate that here to bypass the “Comments to the Author” section, enter your conflict of interest statement in the “Confidential to Editor” section, and submit your "Accept" recommendation.

Reviewer #1: All comments have been addressed

Reviewer #4: (No Response)

2. Is the manuscript technically sound, and do the data support the conclusions?

The manuscript must describe a technically sound piece of scientific research with data that supports the conclusions. Experiments must have been conducted rigorously, with appropriate controls, replication, and sample sizes. The conclusions must be drawn appropriately based on the data presented.

Reviewer #1: Yes

Reviewer #4: Yes

3. Has the statistical analysis been performed appropriately and rigorously?

Reviewer #1: Yes

Reviewer #4: Yes

4. Have the authors made all data underlying the findings in their manuscript fully available?

The [PLOS Data policy](https://eur01.safelinks.protection.outlook.com/?url=http%3A%2F%2Ftrack.editorialmanager.com%2FCL0%2Fhttp%3A%252F%252Fwww.plosone.org%252Fstatic%252Fpolicies.action%2523sharing%2F1%2F010f01932bfbf515-50a35f71-ecd8-4fa8-beea-51ad2d7906e9-000000%2FPwdfRcVKp656m28kBHLLFBC_65RtlLqost4qdtnz2AQ%3D185&data=05%7C02%7Chelena.wallin%40ki.se%7Ca7887eabd8414a5942e008dd04dc1aa1%7Cbff7eef1cf4b4f32be3da1dda043c05d%7C0%7C0%7C638672065581087201%7CUnknown%7CTWFpbGZsb3d8eyJFbXB0eU1hcGkiOnRydWUsIlYiOiIwLjAuMDAwMCIsIlAiOiJXaW4zMiIsIkFOIjoiTWFpbCIsIldUIjoyfQ%3D%3D%7C0%7C%7C%7C&sdata=z%2FTeIErZANfZCBEu%2Fcz9etosRlWjGM3q25Y1ldu5RJM%3D&reserved=0) requires authors to make all data underlying the findings described in their manuscript fully available without restriction, with rare exception (please refer to the Data Availability Statement in the manuscript PDF file). The data should be provided as part of the manuscript or its supporting information, or deposited to a public repository. For example, in addition to summary statistics, the data points behind means, medians and variance measures should be available. If there are restrictions on publicly sharing data—e.g. participant privacy or use of data from a third party—those must be specified.

Reviewer #1: Yes

Reviewer #4: Yes

5. Is the manuscript presented in an intelligible fashion and written in standard English?

PLOS ONE does not copyedit accepted manuscripts, so the language in submitted articles must be clear, correct, and unambiguous. Any typographical or grammatical errors should be corrected at revision, so please note any specific errors here.

Reviewer #1: Yes

Reviewer #4: Yes

**6. Review Comments to the Author**

Please use the space provided to explain your answers to the questions above. You may also include additional comments for the author, including concerns about dual publication, research ethics, or publication ethics. (Please upload your review as an attachment if it exceeds 20,000 characters)

Reviewer #1: Thank you for thoroughly addressing the comments and revising the manuscript. The changes have clearly improved the article's quality, making it more informative and valuable to the field. I confirm that the manuscript meets the journal's standards for data availability, statistical integrity, and readability.

**Reviewer #4**: Dear Editor,
The original cross-sectional and longitudinal research paper entitled “Kynurenines and aerobic exercise capacity in chronic kidney disease: a cross-sectional and longitudinal study” is well-written, structured and developed by Wallin et al. in suitable English with a clear structure. They implemented a cross-sectional and longitudinal study to evaluate the association between aerobic exercise capacity and the plasma levels of accumulation of tryptophan and kynurenines in chronic kidney disease. They finally found that aerobic exercise capacity is inversely associated with the plasma levels of accumulation of tryptophan and kynurenines in chronic kidney disease. The findings are interesting, the methodology is suitable and novel, and the results have been appropriately discussed. I have two main concerns regarding this manuscript. First, there are several data in Table form such as Tables 3, 4 and 6 which can be considered and assigned as supplementary data. Second, the presentation strategy in this paper is not suitable. The paper is not illustrative. I highly suggest to summery the data in Tables into some appropriate illustration and present them in one or two figures. After addressing these major revisions, I may also re-review the paper.

**Authors Response:**

Thank you for your valuable comments regarding the presentation. We have made several changes to the presentation, which we believe have improved the manuscript:

1) **We have replaced table 2 with six subfigures (Figure 2 a-f below)** which contain the same information as table 2 did but in a more illustrative way. Figure 2 caption can be found on page 11.

**Figure 2 a-f. Box plots of peak workload, TRP, and kynurenines in CKD 2–3, CKD 4–5 and controls at baseline.** Whiskers represent highest through lowest values, boxes the interquartile range and circles individual values. a: p-value by one-way ANOVA with Tukey post hoc test, b-f: p-values by one-way ANOVA with Welch’s test and Dunnett’s T3 post hoc test. KYN = kynurenine, KYNA = kynurenine acid, TRP = tryptophan. Number of subjects: 45-53.

2) **We have added one additional figure - Figure 3 a and b (see below).** This figure replaces the old table 3 and 6, which have been modified and moved to supplementary information, see supplementary table S1 and S2. We think that Figure 3 summarizes the most important findings. Figure 3 caption can be found on page 12.

**Figure 3 a) Association between kynurenines, TRP and aerobic exercise capacity (expressed as peak workload) in CKD 2–5. b) Association between the change in kynurenines, TRP and aerobic exercise capacity over 5 years in CKD 2-3.** *p < 0.05, **p < 0.01, ***, p < 0.001 denotes significant inverse association. Assessed by GLM (generalised linear model) with age, sex and height included in all baseline models., KYN = kynurenine, KYNA = kynurenic acid, TRP = tryptophan, W_max_ = peak workload on maximal exercise test.

3) **We have also constructed one new table (table 2),** which replaces the previous table 4 and 5. Some data have also been removed. We think the data in table 2 should be kept in the main manuscript and not in the supplementary information. *Table 2 can be found on page 13.* Below is the title of table 2.

**Table 2. Aerobic exercise capacity, kynurenines and TRP at baseline and at 5 years follow-up, and a comparison between CKD 2–3 and controls regarding the change in these variables.**

4) **We have changed the figure caption and design of figure 4 (previously figure 2).** *The caption can be found on page 12.*

**Figure 4. The relationship between aerobic exercise capacity expressed as peak workload as a percentage of predicted peak workload and KYN/TRP ratio, divided by CKD 2-3, CKD 4-5 and controls**. KYN = kynurenine, TRP = tryptophan.

5) **We have made several changes in the text in the results section due to the new figures and tables.** They are visible in the tracked changes version of the manuscript. The most important changes are that we added the values of GFR and hemoglobin at baseline and at follow-up for both CKD and controls (*page 294, line 286-291 in tracked changes manuscript)*. This information could be found in a table in the previous version.

***To summarize, our main data are now presented as:***

**Figure 1** – Study population

**Figure 2** - Box plots of peak workload, TRP, and kynurenines in CKD 2–3, CKD 4–5 and controls at baseline

**Figure 3 a) and b).** 3 a) Association between TRP and kynurenines and baseline exercise capacity in CKD 2–5. 3 b) The association between the change in those variables over 5 years.

**Figure 4** – A graph of the relationship between aerobic exercise capacity expressed as achieved workload as a percentage of predicted peak workload and KYN/TRP ratio, divided by CKD 2-3, CKD 4-5 and controls.

**Table 1** - Subject characteristics for the whole study population

**Table 2** - Aerobic exercise capacity, kynurenines and TRP at baseline and at 5 years follow-up, and a comparison between CKD 2–3 and controls regarding the change in these variables.

**Supporting information:**

S1 table: Association between aerobic exercise capacity and TRP, kynurenines, TRP and GFR in CKD 2–5 at baseline (details from the generalised linear models).

S2 table: Predictors of the change in aerobic exercise capacity from baseline to 5-year follow-up in CKD 2–3 ((details from the generalised linear models).

**General comment from authors to reviewers and editor:**

We have removed the supplementary file that contained analyses on subjects lost to follow-up and instead added in the manuscript that p-values were > 0.05 (page 14, line 294-296 in tracked changes manuscript) for differences between subjects attending follow-up and subjects lost to follow-up. We have also removed a supplementary file that contained detailed output from GLMs, as we do not think the information need to be provided there. They are available upon request.

We hope that you find that the adjustments have improved the manuscript and thank you for reviewing it.

Kind Regards,

Helena Wallin, main author
